# Compressive Strength Evaluation of Ultra-High-Strength Concrete by Machine Learning

**DOI:** 10.3390/ma15103523

**Published:** 2022-05-13

**Authors:** Zhongjie Shen, Ahmed Farouk Deifalla, Paweł Kamiński, Artur Dyczko

**Affiliations:** 1 Xijing University, Xi’an 710123, China; 2 Structural Engineering and Construction Management Department, Faculty of Engineering and Technology, Future University in Egypt, Cairo 11835, Egypt; 3 Faculty of Civil Engineering and Resource Management, AGH University of Science and Technology, Mickiewicza 30, 30-059 Kraków, Poland; pkamin@agh.edu.pl; 4 Mineral and Energy Economy Research Institute of the Polish Academy of Sciences, J. Wybickiego 7a, 31-261 Kraków, Poland; arturdyczko@min-pan.krakow.pl

**Keywords:** UHSC, building material, compressive strength, soft computing technique, concrete

## Abstract

In civil engineering, ultra-high-strength concrete (UHSC) is a useful and efficient building material. To save money and time in the construction sector, soft computing approaches have been used to estimate concrete properties. As a result, the current work used sophisticated soft computing techniques to estimate the compressive strength of UHSC. In this study, XGBoost, AdaBoost, and Bagging were the employed soft computing techniques. The variables taken into account included cement content, fly ash, silica fume and silicate content, sand and water content, superplasticizer content, steel fiber, steel fiber aspect ratio, and curing time. The algorithm performance was evaluated using statistical metrics, such as the mean absolute error (MAE), root mean square error (RMSE), and coefficient of determination (R^2^). The model’s performance was then evaluated statistically. The XGBoost soft computing technique, with a higher R^2^ (0.90) and low errors, was more accurate than the other algorithms, which had a lower R^2^. The compressive strength of UHSC can be predicted using the XGBoost soft computing technique. The SHapley Additive exPlanations (SHAP) analysis showed that curing time had the highest positive influence on UHSC compressive strength. Thus, scholars will be able to quickly and effectively determine the compressive strength of UHSC using this study’s findings.

## 1. Introduction

Ultra-high-strength concrete (UHSC) is becoming increasingly popular because of its superior mechanical qualities, increased ductility, and longer life expectancy [1]. If UHSC is cured for 28 days, it will have a compressive strength of more than 120 MPA, even after cracking. To attain excellent characteristics, UHSC’s maximum density was carefully designed. Particle packing density in UHSC results in low permeability and dense microstructures [2,3]. Due to the inclusion of distributed discrete fibers, UHSC has increased mechanical strength and crack resistance. There are a variety of civil engineering applications using UHSC, which range from building to rehabilitation to repair. The mechanical characteristics of UHSC are now being evaluated in current practice, by conducting complete experimental examinations. Experimental approaches can be used to determine the precise link between material qualities and mix design, but this requires a significant investment, in terms of both time and money [4]. Variables for UHSC include the cement content, the water content, the additive material content, the fiber content (e.g., steel fibers), the content and type of admixtures, and aggregates content and type (e.g., superplasticizer) [5,6,7]. The addition of dispersed short-discrete fibers to concrete increased crack resistance and improved mechanical characteristics [8,9,10,11,12,13,14,15,16]. Steel fibers are employed to increase the toughness and post-cracking behavior of the cementitious material [17,18,19,20]. Despite several experimental studies in the literature, it is still difficult to predict the characteristics of UHSCs containing various mixtures of components using computational methodologies. Thus, in this work, an attempt was made to anticipate the compressive property of UHSC using soft computing techniques.

Complex issues in a variety of engineering domains can be effectively solved using soft computing approaches. Machine learning (ML) approaches may be used to predict the final output after being provided an input data set. In order to forecast the characteristics of concrete, two ML strategies were used, i.e., a standalone approach (based on a single model) and an ensemble approach (such as AdaBoost and bagging). Ensemble models beat individual ML models in terms of performance, according to studies. However, there are examples of ML models that may be used to predict cement composites characteristics. There has been a detailed evaluation of the use of ML approaches to anticipate concrete mechanical characteristics [21]. In addition, a number of studies have been done to predict the mechanical characteristics of different types of concretes, such as high-performance concrete (HPC) [22], self-healing concrete [23], recycled aggregate concrete (RCA) [24], phase change material-integrated concrete [25], etc. Han, et al. [26] employed a machine learning technique to forecast HPC compressive strength. Cement, fine aggregates, FA, GGBFS, coarse aggregates, age, water, and five other combination variables were included in the dataset’s input parameters. The compressive strength of HPC was accurately predicted by the established model. This article forecasts the compressive strength of UHSC using soft computing techniques and will serve as a baseline to save time and money for future researchers.

The previous studies were related to high performance concrete with a compressive strength around 10–80 MPa [27]. However, this study is related to ultra-high strength concrete (UHSC) with a compressive strength of 100–160 MPa, where the particle packing theory is important. Additionally, the effect of raw ingredients on compressive strength was not investigated by previous studies, which remains a research gap. Therefore, the effect of input parameters (raw materials) on the output parameter (compressive strength) was evaluated using SHapley Additive exPlanations and their interaction was explained. The compressive strength of UHSC may also be predicted using machine learning methods in an alternate approach, to save experimental time and money. In this paper, a variety of ensembled machine learning approaches were used to estimate the compressive of UHSC. XGBoost, AdaBoost, and Bagging are included as ensemble machine learning models. In addition, all models were tested using statistical criteria, and a comparison was made between several machine learning models. A better model was then proposed based on the performance of several statistical parameters to predict UHSC outcomes. Furthermore, a post hoc model-agnostic technique, i.e., SHapley Additive exPlanations (SHAP), was also implemented to give ML model insight [28,29]. The integration of SHAP with ML algorithms was performed in the current research to provide a comprehensive understanding of the mix design of concrete, regarding its strength parameters through its non-linear complex behavior, and to describe the contribution of input parameters by assigning a weight factor to each input parameter. This will be highly beneficial for the development of durable and sustainable concrete mixes.

## 2. Soft Computing Techniques

In order to get the best results, ensemble learning trains numerous base learners to aggregate their findings according to a predetermined methodology [30]. The design and building of fundamental learners, as well as their integration, is critical to ensemble learning algorithms. Based on how base learners collaborate, ensemble learning may be divided into parallel and sequential forms. No substantial connections between individual learners can be found in the parallel ensemble, as demonstrated by the bagging technique. Learners in a sequential ensemble are highly interdependent and sequentially formed, as shown by boosting [31]. Here, the fundamentals of ensemble approaches are briefly discussed.

**Figure 1:**
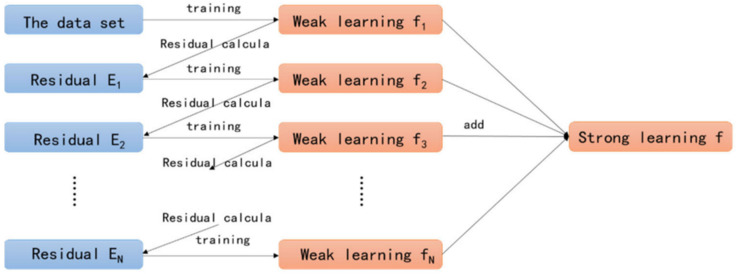
Structure of XGBoost algorithm [32].

Iteratively updating the previous classifier’s parameters reduces the gradient of the loss function and generates a new classifier. The regression tree group is assured to have the highest generalization ability by minimizing the error of prediction across numerous regression trees. The loss function of the model is enhanced by including the regular term. As part of this process, a Taylor expansion of the loss function is used to calculate the split node. The performance of generalization and computation has been enhanced by the use of the regularization approach and second-order derivative information [32]. Figure 1 shows the XGBoost algorithm’s structure.

**Figure 2:**
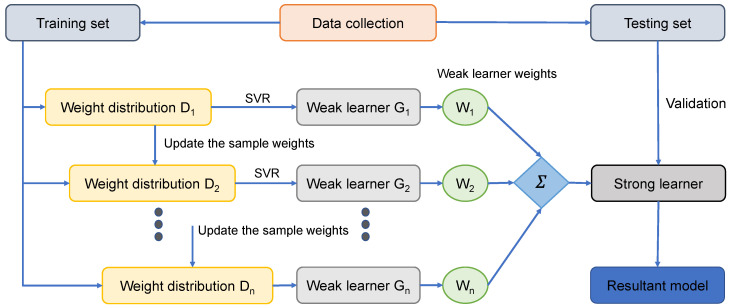
AdaBoost algorithm [33].

A sequential ensemble may be built using the boosting approach. It creates a mediocre learner based on the first set of data. After that, a new weak learner is created to try to correct the mistakes of the previous weak learner. To approach the final prediction model, all weak learners must be included into it. All samples are given equal weight when AdaBoost is used to start the dataset. When a new learner makes a mistake, the samples that it gains weight on, obtain the weight that the first learner gets right. This process has a predetermined number of repetitions, before an error occurs. Updating the training sample weights in subsequent rounds takes the weaker learners’ performance into account. Figure 2 depicts an ensembled support vector regressor technique with AdaBoost.

**Figure 3:**
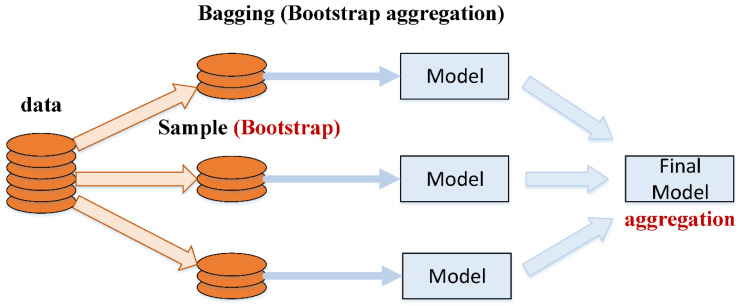
Process flow of bagging algorithm [34].

Bootstrapping and aggregation are two parts of the process of bagging. Training several models is made possible by regularly dividing the full dataset into smaller groups (base learners). The final forecast is the sum of the individual model results. These estimations are averaged together to obtain this forecast in the regression example. According to the categorization example, the voting process is used to make a final forecast. Algorithms such as support vector regressor, adaptive boosting, and bagging were used in this work to predict concrete properties, all of which have been demonstrated to perform well in previous studies for normal strength concrete. The process flow of the bagging algorithm is shown in Figure 3.

## 3. Interpretability of Model Using SHAP

The establishment of a robust prediction tool is gaining attention due to the ML models learning ability from recognized data and for prediction responses in unknown areas. However, lower interpretability and greater complexity is common in most machine learning modelling approaches [35]. SHAP is derived from game theory Shapley values [36]. Its employment is intended to determine the importance of different features within models [35,37]. In SHAP, the feature importance (*j*) for model outcome f; $\phi^{j}\left(f\right)$, is allotted weight for feature contribution summation towards output of model $f\left(x_{i}\right)$ for the overall potential combinations of features [38]. The expression for $\phi^{j}\left(f\right)$ is shown in Equation (1), as given below:

(1) $$\phi^{j}\left(f\right)=\sum_{S\subseteq \left\{x^{1},\ldots \ldots ,\;\;\;x^{p}\right\}/\left\{x^{j}\right\}}\frac{\left|S\right|!\left(p-\left|S\right|-1\right)!}{p!}\left(f\left(S⊔\left\{x^{j}\right\}\right)-f\left(S\right)\right)$$

where; *S* = features subset, *p* = feature number in model, and $x_{j}$ = feature *j*. 

In the SHAP process, the importance of a feature is investigated by quantifying the prediction errors when disturbing a specified value of a feature. The prediction error sensitivity is considered for assigning a weight to feature importance, while perturbing its value. The trained ML model performance is also explained by using SHAP. SHAP uses an additional feature attribution method, i.e., linear input factor addition, to explain an interpretable model, is taken by the model output. As an illustration, a model having input factors $x_{i}$; where i ranges from 1 to k, and; k represents input factors number and *h* ($x_{s}$), shows an explanation model having $x_{s}$ as a simplified input, whereas; Equation (2) is implemented to present an original model f(x):

(2) $$f\left(x\right)=h\left(x_{s}\right)=\emptyset_{0}+\sum_{i=1}^{p}\emptyset_{i}x_{s}^{i}$$

where $\emptyset_{0}$= constant without any information (i.e., no input), and p = input feature number.

**Figure 4:**
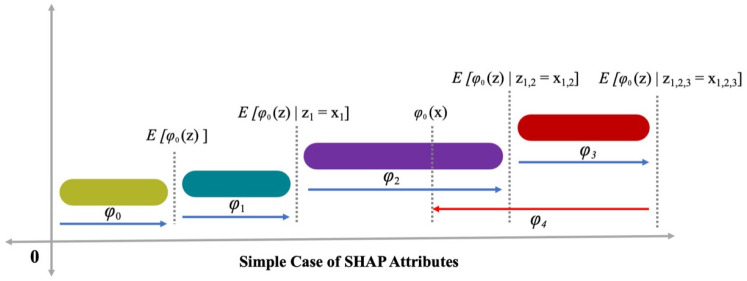
Attributes of SHAP.

The mapping function, i.e., $x=m_{x}\left(x_{s}\right)$, has a correlation with both x and $x_{s}$ inputs. Lundberg and Lee [35] explained Equation (9), in which the prediction value, i.e., (*h* ()) is improved by $\emptyset_{0},\;\emptyset_{1},\;\mathrm{and}\;\emptyset_{3}$ terms and a decline of $\emptyset_{4}$ in *h* () value is also noted (Figure 4). There is a single value solution to Equation (9) that includes three preferred properties, i.e., missingness, consistency, and local accuracy. In missingness, it is ensured that no value for importance is assigned to the missing features, i.e., $\emptyset_{i}=0$ is employed by $x_{s}^{i}=0$. Consistency ensures no reduction in attribution, assigned to the respective features, as a change in feature with more impact. In local accuracy, it is ensured that the summation of feature attribution is taken as a function for the outcome, which includes a requirement for the model to match the outcome f for $x_{s}$ as a simplified input. $x=m_{x}x_{s}$ represents the attainment of local accuracy. 

## 4. Data Set

**Figure 5:**
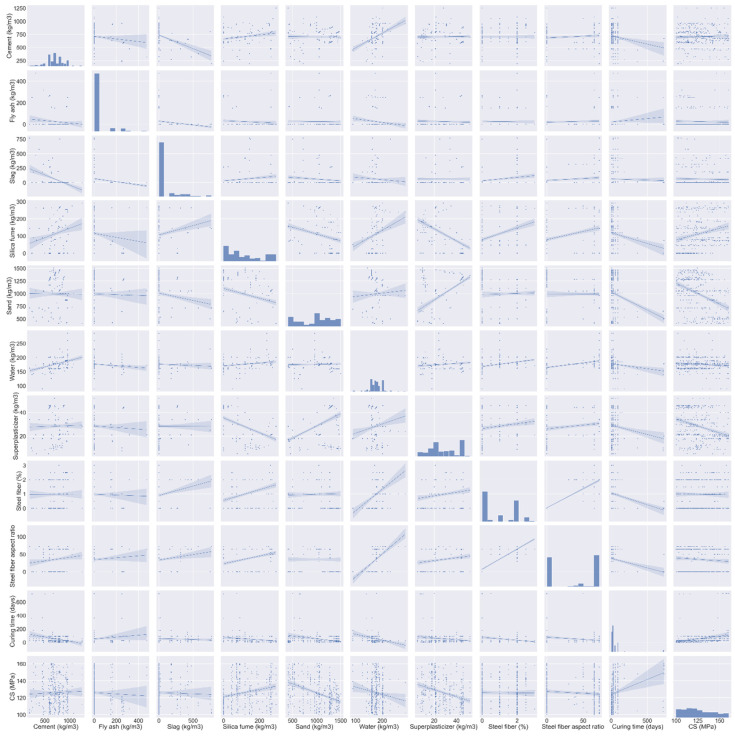
Data description of parameters.

**Figure 6:**
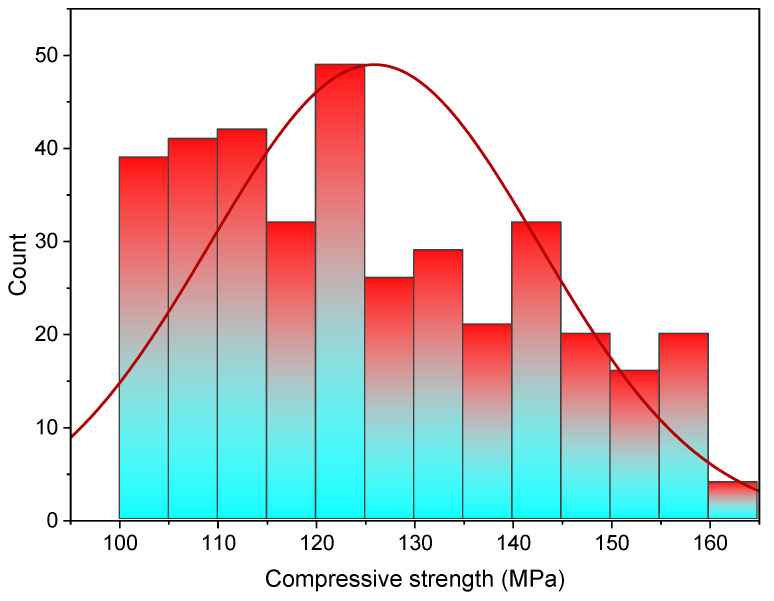
UHSC compressive strength distribution.

Figure 5 shows the data set utilized to forecast UHSC’s compressive strength. The literature [39] provides a compressive database and there were 372 mix proportions with 10 input parameters selected from the data in the range of 100–160 MPa. These include cement content, fly ash, and silica fume content, as well as sand and water. Input parameters of steel fiber aspect ratio and curing time are also included. Predictor variables of the output parameter (compressive strength) are based on these input parameters. Each variable’s range and lowest and maximum values are shown in Figure 5. There is also a figure that presents the mean and standard deviation for each variable. Compressive strength was predicted using Anaconda software’s Spyder and Python scripting. The histogram of compressive strength used in this study is shown in Figure 6.

## 5. Results and Discussion

### 5.1. XGBoost

**Figure 7:**
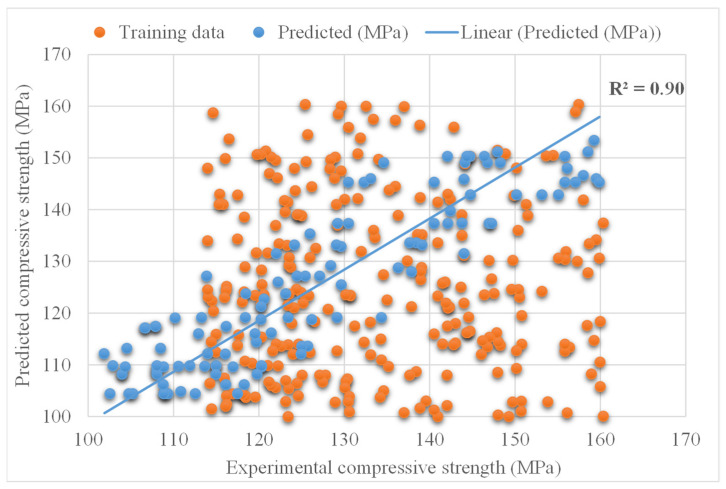
Experimental and XGBoost predicted results.

**Figure 8:**
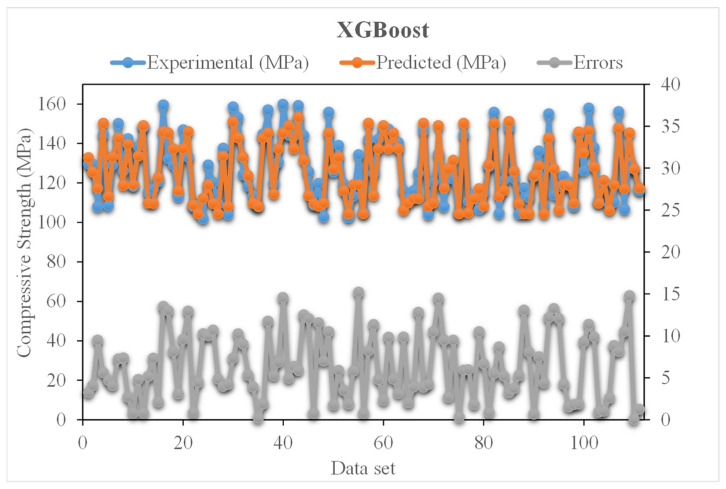
Experimental and SVR predicted values with errors.

The comparison of experimental and predicted values with the XGBoost algorithm for compressive strength of UHSC is presented in Figure 7. The XGBoost exhibited reasonable predicted results, with low variation for the compressive strength of UHSC. An acceptable R^2^ value of 0.89 shows the suitability of the XGBoost model. Figure 8 illustrates the error distribution of the experimental and XGBoost predicted values of compressive strength for UHSC. The average values of error for compressive strength are 6.42 MPa. Whereas 50% of error values are less than 5 MPa, 37% are from 5 to 10 MPa, and 24% are higher than 10 MPa. 

### 5.2. AdaBoost

**Figure 9:**
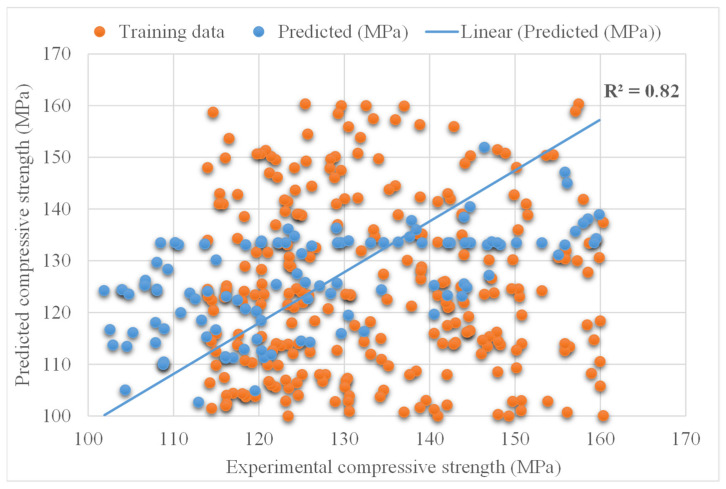
Experimental and AdaBoost predicted results.

**Figure 10:**
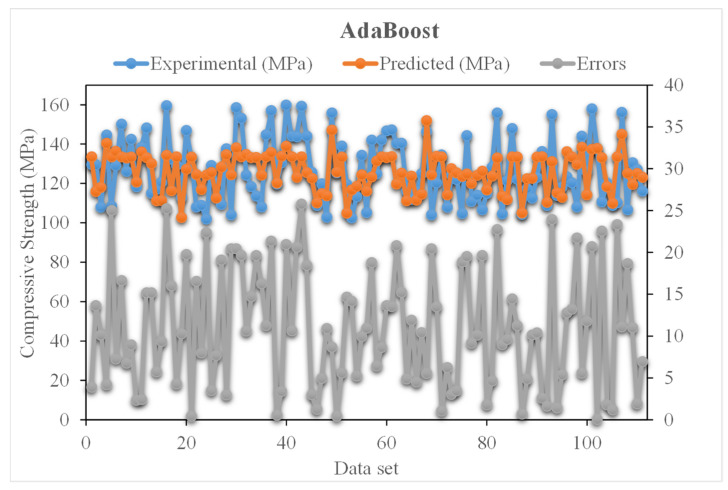
Experimental and AdaBoost predicted values with errors.

Figure 9 shows the experimental and predicted AdaBoost algorithm results for compressive strength of UHSC. The R^2^ value for AdaBoost is 0.82 and represents less precise results than that of the XGBoost algorithm. The distribution of experimental and Adaboost predicted values with errors for compressive strength of UHSC is demonstrated in Figure 10. It is noted that 30% of error data is below 5 MPa, 29% is from 5 to 10 MPa, and 52% is higher than 10 MPa. The lower error and higher R^2^ value indicated the better accuracy of XGBoost model compared to AdaBoost. 

### 5.3. Bagging

**Figure 11:**
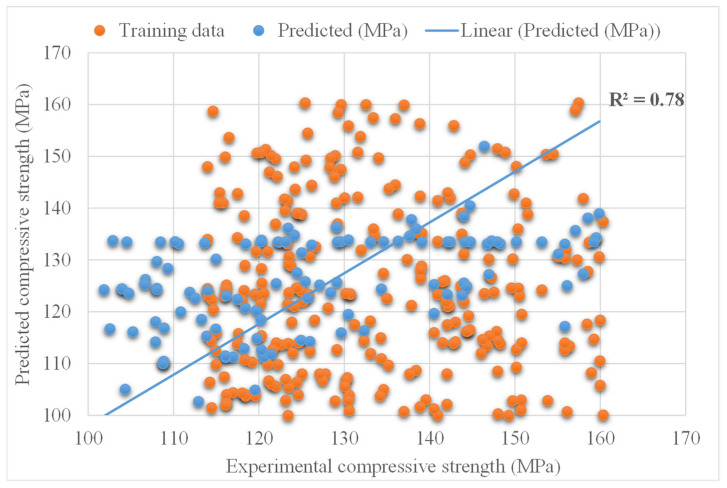
Experimental and BSA predicted results.

**Figure 12:**
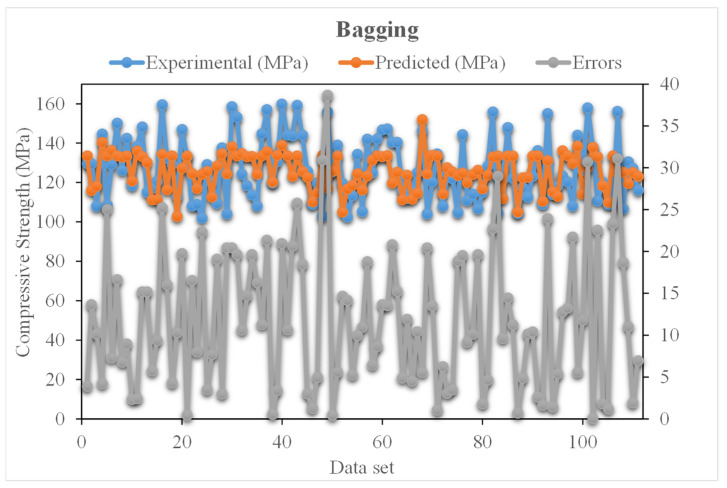
Distribution of experimental and BSA predicted values with errors.

The experimental and bagging predicted results of UHSC for compressive strength are shown in Figure 11. The R^2^ for this model is 0.78, which shows less suitable results compared to the above two models. However, the predicted compressive strength results of UHSC for XGBoost are better than the other ensembled models. Figure 12 demonstrates the distribution of experimental and bagging predicted values with errors for compressive strength of UHSC. Whereas 30% of error values are below 5 MPa, 17% of values range from 5 to 10 MPa, and 62% of values are found above 10 MPa. The error and R^2^ values for the compressive strength of UHSC for XGBoost are more accurate than the bagging model. Wang, et al. [33] reported that the AdaBoost machine learning approaches predicted the best compressive strength of geopolymer composites. Zhu, et al. [40] used machine learning to forecast the splitting tensile strength (STS) of concrete containing recycled aggregate (RA) and revealed that the precision level of the bagging model was better. Ahmad, et al. [41] studied the boosting and AdaBoost ML approaches to predict the compressive strength of a high-calcium fly-ash-based geopolymer. Bagging indicated the best results. However, the R^2^ and error values obtained for the XGBoost ensemble machine learning models are acceptable. Thus, this finding implies that XGBoost could predict outcomes with a higher degree of accuracy than the other models.

### 5.4. Comparison of All Models

**Figure 13:**
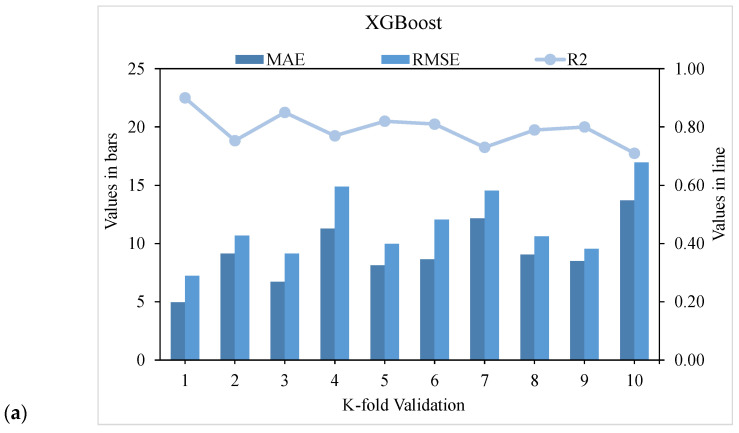

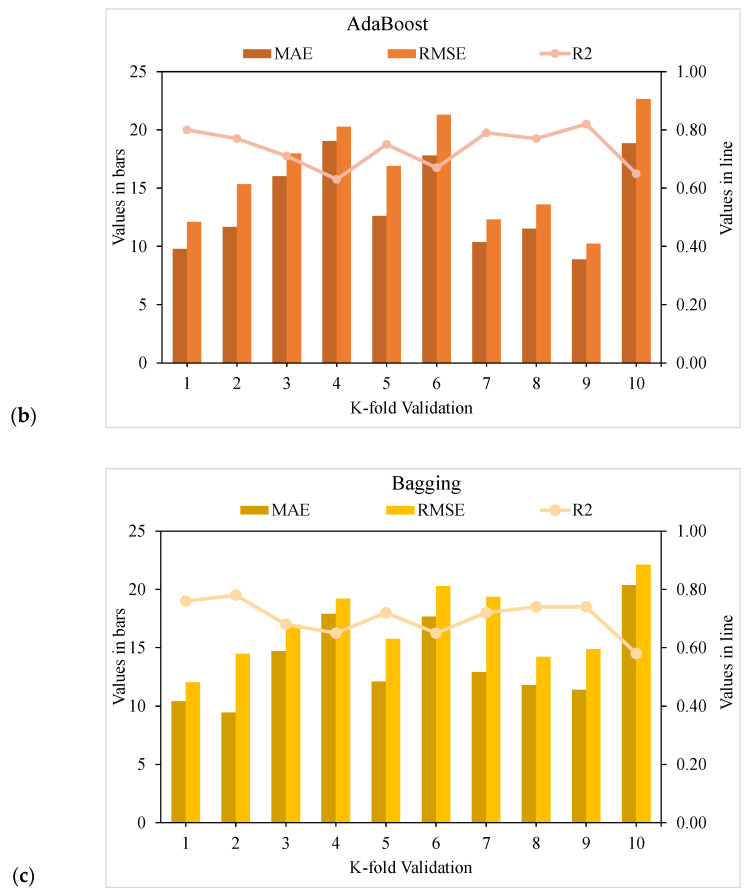
Statistical representation: (**a**) XGBoost; (**b**) AdaBoost; (**c**) Bagging.

The validity of a model during execution is assessed by employing the K-fold cross-validation method. Statistical checks are used to evaluate the performance of models [42,43,44,45]. Usually, random dispersion is performed by splitting data into ten groups for k-fold cross-validation, and this process is repeated ten times to obtain acceptable results. Table 1 shows statistical checks for all models. The R^2^ values for the XGBoost, AdaBoost, and Bagging models were 0.90, 0.82, and 0.78, respectively, as shown in Figure 13a–c. It was found that the R^2^ of XGBoost was higher than that of all other models, with low error values for the compressive strength of UHSC. 

**Figure 14:**
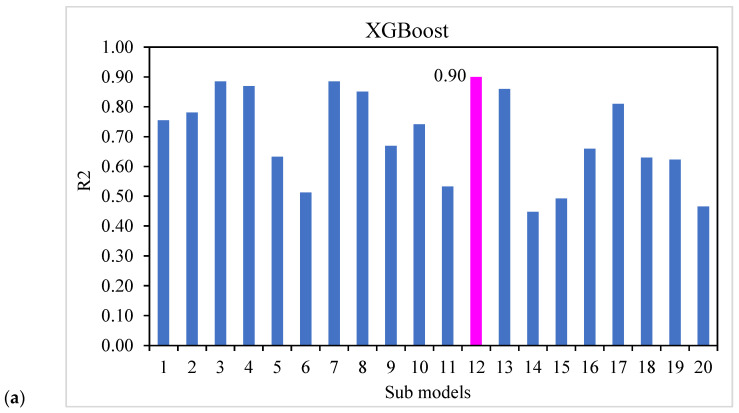

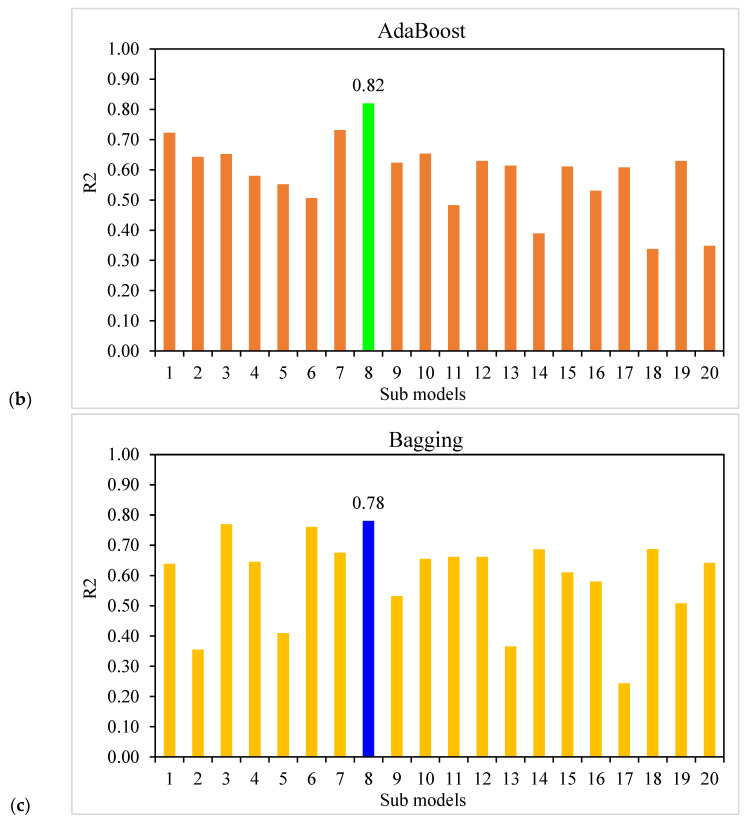
Sub-model results: (**a**) XGBoost; (**b**) AdaBoost; (**c**) Bagging.

The compressive strength of UHSC was predicted using ensembles of machine learning approaches in this work, which aimed to provide efficient and reliable findings. With an R^2^ value of 0.90, XGBoost’s output provided more exact predictions for UHSC compressive strength. Using an optimized model from the 20 sub-models shown in Figure 14a–c to predict compressive strength, the XGBoost ensemble machine learning models performed better. It was, thus, shown that, compared to the other models, the XGBoost ensembled models demonstrated an excellent accuracy and low error.

Mahjoubi, et al. [46] constructed an auto-tune learning framework for ultra-high-performance concrete flowability, mechanical characteristics, and porosity prediction (UHPC). Other models were also considered by Mahjoubi et al. [47,48] in previous studies for multiple functions, and can be applied to similar types of studies in the future. This study evaluated compressive strength in the range of 100–160 MPa, considering 372 mix proportions with 10 input parameters selected from the database of Mahjoubi et al. [39,46]. A much more relevant model could be obtained by increasing the number of datasheets and by importing a significantly higher number of mixtures, as well as by considering higher input parameters. Therefore, it is suggested that the number of data points and outcomes in future investigations be raised by experimental work, field tests, and numerical analysis, using a range of approaches (e.g., Monte Carlo simulation, among others). Environmental factors (such as high temperatures and humidity) could be included in the input parameters, along with a detailed explanation of the raw materials, to improve the models’ responses. 

## 6. Enhanced Explainability of ML Models

**Figure 15:**
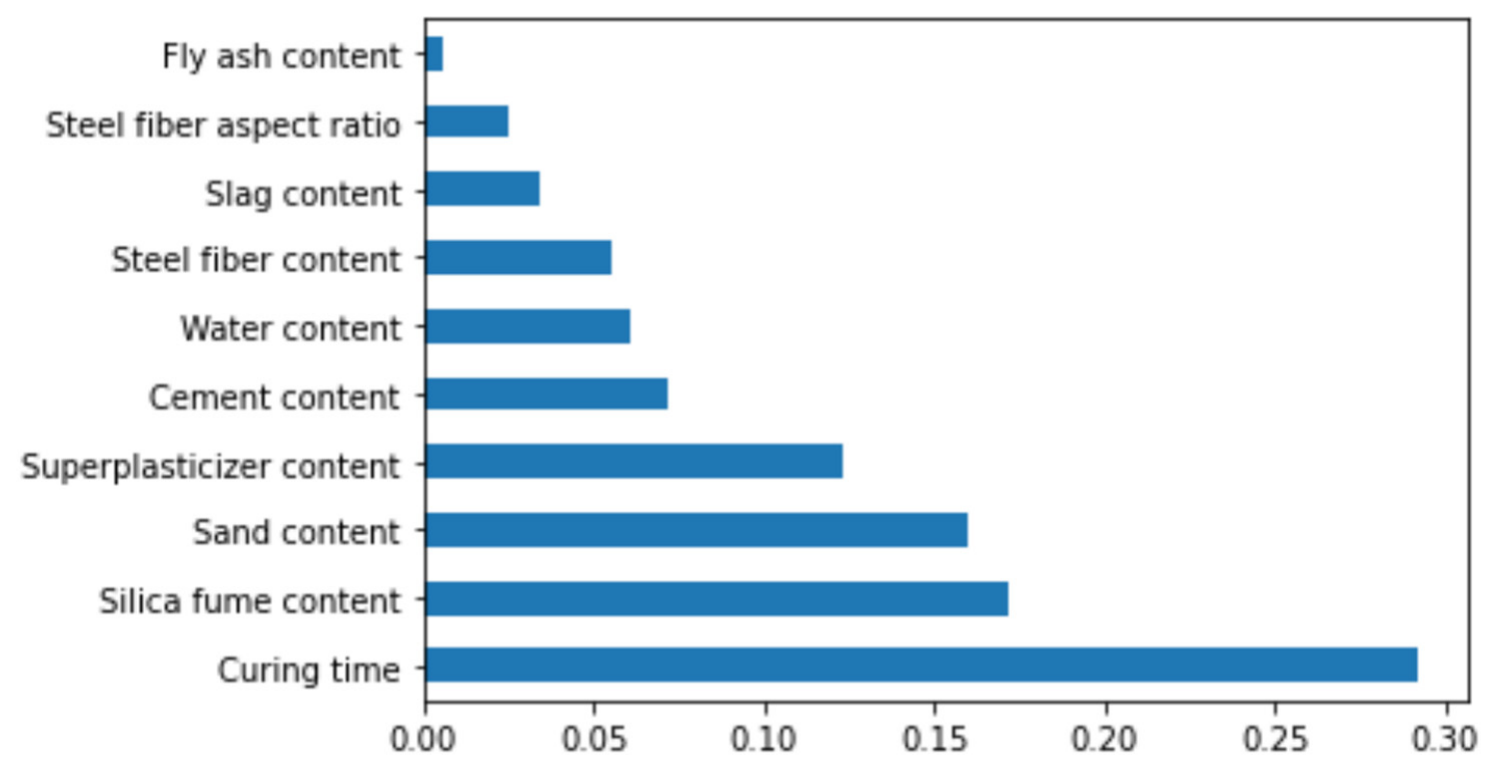
Feature importance.

In the current research, an in-depth description of the ML model and dependencies/interactions of all the considered features is provided. Initially, by implementing the SHAP tree explainer for the entire dataset, an enhanced global representation of feature influences, by merging local explanations from SHAP, is provided. A tree-like SHAP approximation technique, named TreeExplainer, was employed [49]. In this technique, the internal structure of tree-based models was evaluated; that is the sum of calculations set having a linkage with the leaf node of a tree model that led to low-order complexity [49]. The XGBoost model denotes the performance forecasting with higher precision for compressive strength of ultra-high strength concrete (UHSC), so in the current section, the model’s interpretation is done for compressive strength of UHSC using SHAP. The correlation of various features with SHAP values for compressive strength of UHSC (as obtained from the XGBoost ensemble modelling) is presented in Figure 15. 

It can be noted here that the curing time has highest SHAP value in the case of compressive strength prediction for UHSC. Increasing curing time would result in greater compressive strength, as UHSC has a high quantity of binders, i.e., silica fume, slag, fly-ash etc., so the hydration process requires more curing time, ultimately resulting in enhanced compressive strength. The silica fume content feature, i.e., a key parameter of UHSC and directly influencing the compressive strength, has the second highest SHAP value. Subsequently, sand is the third most influential feature, as shown in Figure 15. In UHSC, particle packing density would be difficult to achieve in the case of higher sand contents. Super-plasticizer is fourth in the row, due to its higher SHAP value. More super-plasticizer and a lesser water content positively influences the compressive strength of UHSC. Similarly, the influence of cement is next in terms of SHAP value, followed by the water, steel fiber, and fly-ash features. All these features have their unique roles in the compressive strength of UHSC. Fly ash has little effect on compressive strength and influences the workability of UHSC more.

**Figure 16:**
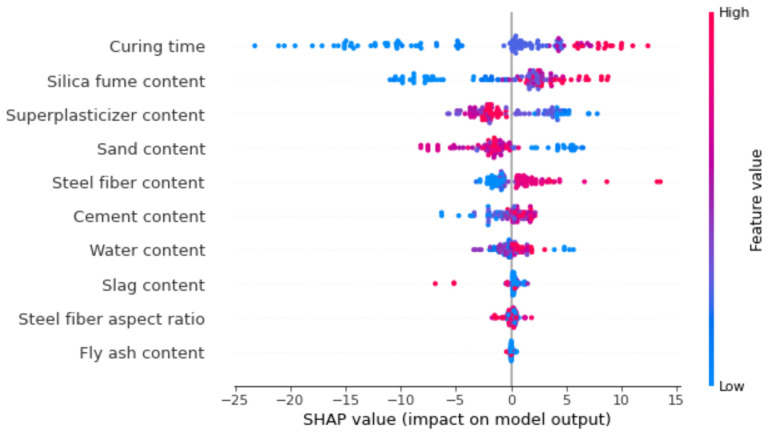
SHAP plot.

Figure 16 depicts the violin plot SHAP values for all the corresponding features that were considered to predict the compressive strength of UHSC. In the said plot, a unique color represents every feature’s value and the corresponding SHAP value at the x-axis represents the outcome contribution. For instance, for curing time and silica fume content as input features, a positive influence can be observed from the right side of the axis, showing a direct relationship for both the features with the compressive strength of UHSC. A SHAP value of almost 14, in the form of red points at the rightmost, shows that a higher curing time enhances the UHSC compressive strength. However, in case of the super-plasticizer feature, a positive influence is seen, but only up to the optimal content. Beyond this content, it has a negative influence, in the form of a blue color (i.e., lower values). It is usually observed that upon enhancing the water-binder ratio, the compressive strength tends to increase up to a certain limit, and then further enhancement of the water-binder ratio decreases the compressive strength. In the same manner, a higher quantity of sand negatively influences the compressive strength of UHSC, as its particle packing is disturbed. Furthermore, a weaker bond would be observed in the case of a higher sand content with respect to binder. Steel fiber and cement content also show a positive influence. Last, water has both positive and negative influences and is directly related to the binder content. A higher water content would result in a reduced UHSC compressive strength. Fly ash and slag, although they do not have a considerable impact on compressive strength of UHSC, still display more or less similar feature influences. These observations are dependent on the database used in this study, and results with greater accuracy may be acquired in the case of more data points.

**Figure 17:**
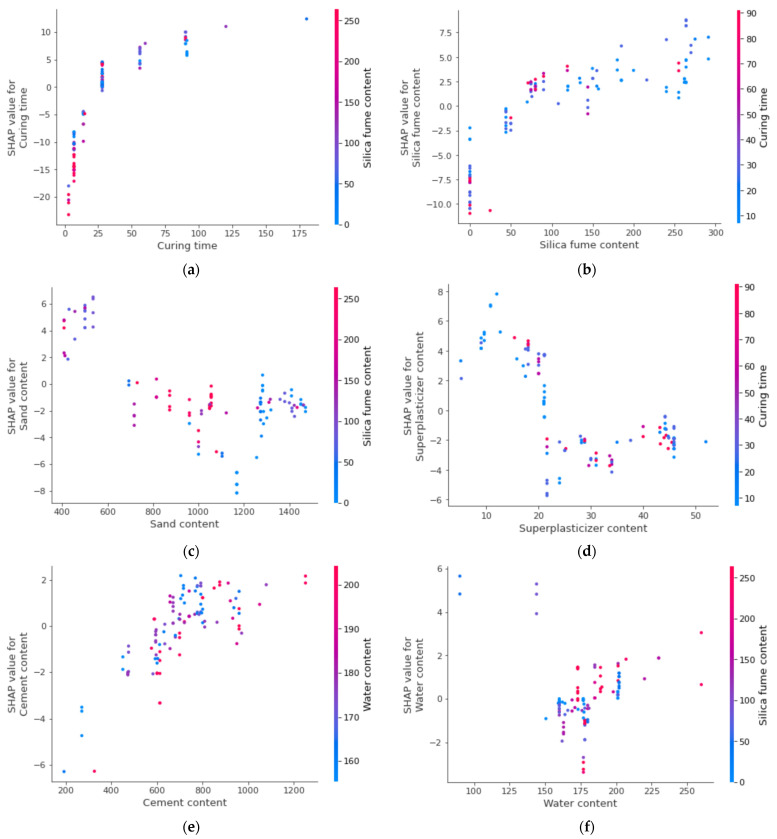
Interaction plot of various parameters: (**a**) Curing time; (**b**) Silica fume content; (**c**) Sand content; (**d**) Superplasticizer; (**e**) Cement content; (**f**) Water content.

The interaction of the various considered features with the compressive strength of UHSC is presented in Figure 17. The curing feature interaction is shown in Figure 17a. It may be observed from the plot that curing time is a major influence of the compressive strength of UHSC and is in a positive/direct relationship. In this scenario, the maximum interaction of curing is with silica fume, hence, aiding in the enhancement of UHSC strength. In Figure 17b, a positive influence of silica on the compressive strength of UHSC is observed. A greater interaction of silica is found with curing time and it is negatively influential. The fine aggregate/sand feature interaction is plot in Figure 17c. The sand content has a negative influence, due to its effect on silica fume. Therefore, the effect of sand on silica fume results in decreased compressive strength. Then, in a row, super-plasticizer shows both positive and negative interactions, depending upon the content (Figure 17d). A lesser content, up to the optimum content, would result in a positive interaction and vice versa. In the same manner, cement content positively interacts and greatly influences the water content, as the w/c ratio has a major role in the development of strength, due to multiple factors, including the hydration process (Figure 17e). In Figure 17f, the interaction of silica fume with water content is shown. The lesser surface area of silica fume demands a higher water content. Furthermore, during pozzolanic activity in the hydration reaction, silica fume needs more water; therefore, a higher interaction of silica fume with water content is observed. 

## 7. Conclusions

Soft computing has recently been employed in the construction sector to forecast the mechanical characteristics of concrete, which has gained the attention of the industry. It was the goal of this study to evaluate the accuracy of soft computing approaches for predicting the compressive strength of UHSC. Ten input variables were used for estimation: i.e., cement content, fly ash, silica fume and silicate content, sand and water content, superplasticizer content, steel fiber, steel fiber aspect ratio, and curing time. As a result of our research, we have come to the following conclusions:

- As evidenced by the R^2^ value of 0.90, the XGBoost method was able to accurately estimate the compressive strength of UHSC from its actual data. However, the ensembled machine learning models, i.e., AdaBoost and Bagging with R^2^ values of 0.82 and 0.78, predicted unacceptable findings for the compressive strength of UHSC.
- A total of twenty sub-models, ranging from 10 to 200 estimators, were utilized to optimize the anticipated compressive strength of UHSC. An ensembled model XGBoost was able to accurately forecast the compressive strength more effectively than the other models.
- XGBoost models demonstrated lower MAE and RMSE, with a higher R^2^ value for compressive strength of UHSC, compared to the other model in the k-fold validation results. XGBoost was proven to have the best compressive strength prediction accuracy for UHSC.
- The model’s performance was evaluated using statistical measures such as MAE and RMSE. However, XGBoost projected superior results, with less error and a higher coefficient of determination for evaluating the compressive strength of UHSC.
- The XGBoost is the best method for predicting the compressive strength of UHSC utilizing soft computing approaches.
- Curing time has highest impact on UHSC compressive strength estimation, followed by silica fume, sand and super-plasticizer content, as depicted by SHAP analysis. Whereas, the compressive strength of UHSC with fly ash content is the least influential.
- The feature interaction plot showed that curing time, cement content, and silica fume positively influence UHSC compressive strength.

## Figures and Tables

**Table 1:** Statistical checks of the XGBoost, AdaBoost, and Bagging models.

Techniques	MAE (MPa)	RMSE (MPa)	R^2^
XGBoost	6.4	7.6	0.90
Adaboost	11.0	13.1	0.82
Bagging	11.9	14.6	0.78

## Data Availability

Data will be available on request from corresponding author.
